# Geographic variability of floating kelp recovery after a marine heatwave event in the Salish Sea and adjacent open coast

**DOI:** 10.1371/journal.pone.0336574

**Published:** 2025-12-02

**Authors:** Danielle C. Claar, Helen Berry, Bart Christiaen

**Affiliations:** Nearshore Habitat Program, Washington State Department of Natural Resources, Olympia, Washington, United States of America; Stockholm University, SWEDEN

## Abstract

Floating canopy kelps, foundational species that support productive nearshore marine forests, are threatened by increasingly common and severe marine heatwaves (MHW). We tracked annual changes in kelp canopies and sea surface temperature (SST) before, during, and after the 2014–2016 MHW from the open coast of Washington State, USA into the Salish Sea. We found spatial differences in kelp canopy recovery timelines and in warming magnitude and duration. Estuarine-dominated sub-regions experienced slower recovery (up to three years) than ocean-driven areas (one year), even though all sub-regions experienced coincident declines. Mean SST of the warmest month was the most common predictor of annual canopy abundance (4 of 6 sub-regions), and the best predictor in the two exposed coast sub-regions, while anomaly magnitude and duration were the best predictors in the two innermost sub-regions. These results suggest that there may be substantive differences in kelp stressors and their interactions between estuarine- and oceanic-dominated nearshore ecosystems. However, while statistically significant, these associations were weak, emphasizing the need to understand effects of MHW on canopy kelp in the context of multiple interacting drivers.

## Introduction

Along many temperate and subtropical latitudes, kelp forests form the foundation of nearshore ecosystems [1], providing primary production, nutrient cycling, and habitat for a diverse biota (e.g., [2–4]). However, nearshore ecosystems are threatened by warming temperatures, and marine heatwaves (MHW) cause substantial stress for kelps [5]. MHW have already increased and are projected to accelerate in coming decades [6]. Since loss of kelp canopies can lead to phase shifts (e.g., [7,8]), a pressing question is how, when, or even if, kelp forests will recover from these severe events. Improved understanding of the spatiotemporal effects of warming on kelp canopies is needed to guide conservation and restoration of these foundational species.

MHW significantly impact kelp canopies [9] though in geographically diverse ways difficult to disentangle from other stressors. For example, kelp canopy declines during the 2014−2016 MHW in the northeast Pacific [10,11], triggered, and were exacerbated by, other stressors [8,12,13]. Toward the southern range extent of giant kelp *Macrocystis pyrifera* in the Northern Hemisphere, temperature thresholds are correlated with resistance to canopy loss, but not recovery, suggesting that recovery may have instead been controlled by local environmental or biotic processes such as altered predator-prey dynamics due to sea star wasting disease [14,15]. Although the 2014−2016 MHW accounted for an estimated 36% decline in four species of canopy kelps across ecoregions [16], responses varied geographically, with >90% loss and minimal recovery in northern California (*Nereocystis luetkeana*; [8]), minimal change in Oregon (*N. luetkeana;* [15]), and substantial losses in Baja California, Mexico (*M. pyrifera;* [17]). Along the open coast of Washington, the response to the 2014−2016 MHW was distinct; kelp canopies (*N*. *luetkeana* and *M*. *pyrifera*) declined in 2014 then recovered within one year [18]. However, a notable gap exists in understanding of kelp response within the adjacent Salish Sea. A biologically rich inland sea, the Salish Sea supports numerous species of kelp, along with mammals, birds, fish, invertebrates, and a vital regional economy. The inland fjord system is tightly coupled to open ocean dynamics, yet terrestrial, atmospheric, and riverine conditions interact with ocean conditions to create unique regional conditions and ecological responses to events like the 2014−16 MHW [10].

Previous studies found that canopy kelp responses to the 2014–2016 MHW were best explained by temperature thresholds. For example, giant kelp biomass was non-linearly related to the SST of the warmest month during the 2014–2016 event in California, with temperatures above 23–24°C instigating drastic declines in resistance [14]. Also, because elevated temperatures are often correlated with low nitrogen levels and other factors, temperature is also used as a proxy for co-occurring stressors. Multiple temperature thresholds have been determined for floating kelp species in the northeastern Pacific, including: 14–15°C, an empirical threshold associated with very low nitrate concentrations off the California coast [19], 16–18°C for microscopic stages of *N. luetkeana* [20], 18–20°C for early life history stages of *N. luetkeana* and other kelp species (but not *M. pyrifera*) [21,22], 21°C for physiological damage to *N. luetkeana* blades [23], 24–25°C for *M. pyrifera* [24–26]. However, maximum temperature is only part of the story, and other MHW properties likely influence persistence (e.g., [27]).

The 2014–2016 Pacific MHW [11] provides a case study in variability of warming trajectories, as the magnitude and duration of warming varied across geographic gradients [10]. In this study, we investigated how MHW propagation influenced kelp canopy (*N. luetkeana* and *M. pyrifera*) resistance and recovery trajectories along a geographic gradient from the open coast of Washington, USA, into the Salish Sea between 2011 and 2018. We tested three hypotheses, which are not mutually exclusive: 1) *Maximum temperature* is a significant factor explaining variability in kelp canopy area. Therefore, canopy loss and recovery are related to thermal thresholds. 2) *Temperature anomaly* is a significant factor explaining variability in kelp canopy area. Therefore, larger deviations from average temperatures are related to more loss and longer recovery times. 3) *Accumulated thermal stress* (i.e., number of days with positive SST anomaly) is a significant factor explaining variability in kelp canopy area. Therefore, more time at elevated temperatures is related to more loss and longer recovery.

## Methods

**Fig 1:**
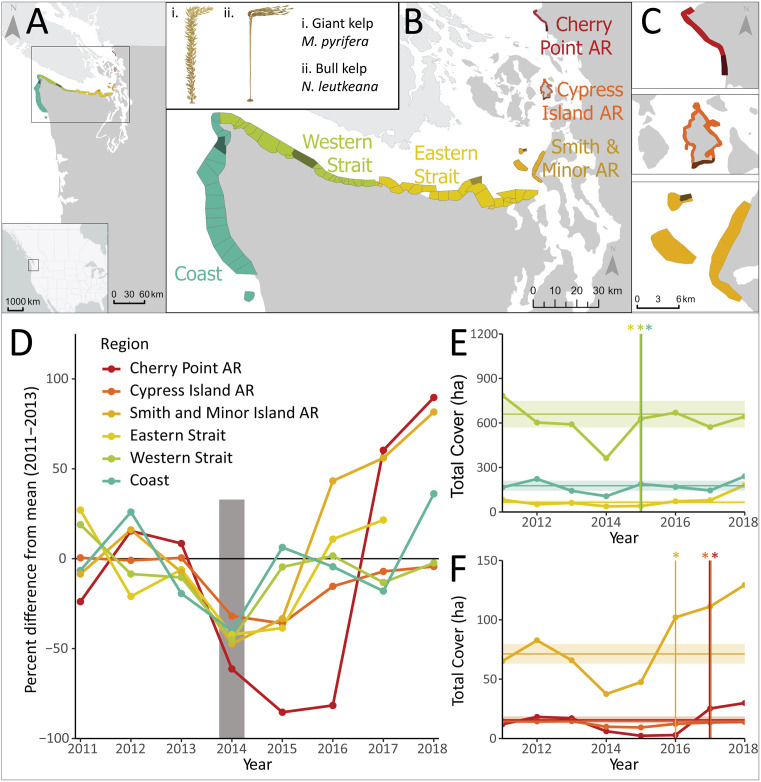
Floating canopy-forming kelp declines and recovery across a geographic gradient in Washington State. A) inset map showing location of study area in the Northeastern Pacific Ocean. B) Washington State (USA) sub-regions included in this study, spanning from the exposed Pacific coast (“Coast”) into the Salish Sea. Map colors correspond to each sub-region, light outlines within sub-regions indicate individual zones in which floating kelp canopy area was tracked, and the darker colored zone in each sub-region corresponds to the location where representative temperature data was extracted for visualization (Fig 2). Inset shows giant kelp (i) and bull kelp (ii) illustrated by Andrea Dingeldein. C) zoomed in view of each Aquatic Reserve. D) Percent difference of floating kelp canopy area compared to a ‘pre-warming’ mean (calculated to include 2011-2013). Lines are colored by sub-region. The gray bar indicates 2014 (the onset of the MHW), and the horizontal black line indicates zero. E) and F) floating kelp canopy area (ha) for the Eastern Strait, Western Strait, and the open coast (E) and the Aquatic Reserves (F). Horizontal colored lines correspond to the mean floating kelp canopy area for each sub-region, and the shaded area corresponds to ±1 s.d. Vertical lines and colored asterisks (*) indicate when each sub-region recovered back to the pre-warming mean. Map credits: Commission for Environmental Cooperation, Statistics Canada 2022.

Annual remotely sensed canopy kelp imagery was collected in Washington State (USA) for 2011–2018 across 6 sub-regions (Fig 1a–c). Kelp canopy area was compared across these sub-regions before, during, and after the 2014–2016 Northeast Pacific MHW. Additionally, data was analyzed for 1989–2021 for the open coast and Strait sub-regions.

### Site description & study species

The study area spans a gradient of physical conditions – from the exposed coast driven by open-ocean processes, through the well-mixed and current-driven Strait, and into a complex network of inner basins that are driven by local patterns of circulation, stratification, and estuarine input.

Along the open coast and western Strait of Juan de Fuca, two canopy-forming species, giant kelp *Macrocystis pyrifera* and bull kelp *Nereocystis luetkeana,* co-occur. In other portions of the Salish Sea, *Nereocystis* is the sole canopy-forming species (S2 Fig). Both species experience large year-to-year variability in population size due to natural climate variations, and abundances tend to covary [28].

The Salish Sea is warming rapidly, with projections of a 1.5–3°C increase by the end of this century [29,30]. Extensive *Nereocystis* losses have been documented in the innermost basin, South Puget Sound [31]. Recently, a statewide indicator identified a distinct pattern of stable populations along the open coast and Strait, losses at some sites, and many sub-basins with insufficient data to identify trends [32].

### Data collection

#### Kelp data.

Each year, imagery was collected from a fixed-wing plane [33,34]. We chose to use a fixed-wing platform to collect this imagery because it provides a balance between large-area survey capability and adequate detectability of kelp canopies. Aerial imagery collection methods are described in Supporting Information, and additional detail is included in Van Wagenen 2015.

Imagery was collected annually from 1989 to 2021 (except 1993) for the open coast and Strait of Juan de Fuca sub-regions, and from 2011–2021 for the DNR Aquatic Reserves (Figs 1a–c and S1; S1 and S2 Tables). For consistency among sub-regions and to limit analyses to a single MHW, only data from 2011–2018 are included in the main analyses. A secondary analysis was conducted for the open coast and Strait for the entire data record (1989–2021). In sub-regions where both species commonly co-occur (i.e., Coast and Western Strait; S2a Fig), the species were considered together (areas of each species were summed to determine total canopy area), as well as separately.

#### Temperature data.

The NOAA satellite-derived sea surface temperature (SST) reanalysis product, *CoralTemp* [35], was used for comparisons with floating kelp canopy area. This product is a nighttime-only measure of sea surface foundation temperature, averaged daily at 5-km resolution. Nighttime-only temperature is used as the basis of the *CoralTemp* product because it provides a stable measurement of temperature and avoids issues with solar glare [35]. The product suite also includes derived products including SST climatology (representing 1985–1990 + 1993) as well as daily SST anomalies (defined as difference in temperature compared to a long-term climatological mean). This dataset provided continuous temperature data across our surveyed area, as well as climatology products that would otherwise not be feasible to determine from shorter-term in situ datasets. Specifically, we used *CoralTemp* to assess three annually summarized temperature metrics: mean daily mean SST of the warmest month, maximum monthly SST anomaly (SSTA; i.e., maximum daily anomaly averaged by month), and annual count of days with SSTA>0°C. Each metric was calculated using approximately the year preceding canopy surveys (e.g., for 2014, September 2013–August 2014). Temperature metrics were extracted for each zone; some zones were close enough that they landed in the same grid cell.

### Data analysis

#### Kelp data.

Kelp canopy area was considered at two spatial scales: 1) sub-regions and 2) zones that subdivided each sub-region into stretches of shoreline with similar geomorphological features such as points, embayments, and sills. As a baseline for comparison, pre-MHW canopy area was calculated as the mean of 2011–2013 (Table 1). This period (2011–2013) was chosen because it was consistently available for all sub-regions and represents “normal” non-heatwave conditions. Although this period does not represent a long-term baseline for kelp canopies, it provides a short-term reference for responses to the MHW. Annual kelp canopy area was then compared as percentage of the pre-MHW mean (Fig 1d), and raw cover data were plotted in Fig 1e and 1f. Next, we calculated mean canopy area for each year (2011–2018). We define recovery as the point when canopy area returned to <1 s.d. of the pre-MHW canopy area. This value was chosen as a quantitative cutoff that allows consistent identification of recovery across sub-regions and years, while accounting for natural interannual variability of kelp canopies. We also calculated variability in canopy area as fold-differences (i.e., the ratio between the lowest and highest canopy area) and as percentages. For models of canopy area and temperature metrics (below), canopy area was scaled to percentage of maximum area observed during the study period. In cases where transformation caused the response variable to be zero or one exactly, we replaced the value with 0.01 or 0.99, respectively. Additionally, we calculated the correlation during 2011–2018 between *Macrocystis* and *Nereocystis* canopy area for the sub-regions where they commonly co-occurred (i.e., open coast and Western Strait), and created density plots to visualize canopy variability (ggridges, [36]; S2b Fig). We tested for differences between *Macrocystis* and *Nereocystis* percentage of maximum canopy area using a Wilcoxon rank-sum test, and for homogeneity of variances using Levene’s test.

**Table 1:** Floating kelp canopy area, variability, and loss in six sub-regions in Washington State, USA, during the main study period (2011-2018) and during the pre-MHW ‘baseline’ (2011-2013). In sub-regions where both *Macrocystis pyrifera* and *Nereocystis luetkeana* were present, their areas were summed.

Region	Min. canopy area (ha) (2011-2018)	Max. canopy area (ha) (2011-2018)	Fold difference of max. relative to min. canopy area (2011-2018)	Mean canopy area ± s.d. (ha) (2011-2018)	Pre-MHW ‘baseline’ mean canopy area ± s.d. (ha) (2011-2013)	Max loss in 2014−16 compared to pre-warming mean
Open Coast	215	441	2.1	335 ± 76	361 ± 63	40%
Western Strait	545	1139	2.1	905 ± 168	978 ± 115	45%
Eastern Strait	90	326	3.6	175 ± 74	154 ± 38	42%
Smith & Minor Island AR	38	129	3.5	80.2 ± 32	71 ± 8	47%
Cypress Island AR	9.3	15	1.6	12.8 ± 2	15 ± 1	36%
Cherry Point AR	2.3	30	13.0	14.2 ± 10	16 ± 3	85%

#### Temperature metrics and kelp canopy responses.

To test for a relationship between warming and kelp canopy loss and recovery, we modeled scaled area at individual zones against three temperature metrics (above). We used R (v4.3.1; [37]) to run a series of beta regression models with a Cauchy latent variable link function (R package *betareg*; [38]) for time periods 2011–2018 (heatwave and adjacent years) and 1989–2021 (entire record for the coast and Strait). We ran these models separately for each sub-region. We estimated explanatory power using pseudo-R^2^. These analysis methods are similar to Cavanaugh et al. (2019), with some modifications due to differences in data sources and hypotheses. First, we assessed kelp canopy area rather than floating kelp biomass. Second, we used maximum SSTA rather than mean SSTA, and we used days with SSTA>0°C instead of heatwave days. Third, to consider variability across sub-regions in temperature trajectories and recovery timelines, we calculated temperature metrics for each year individually rather than collectively between 2014 and 2016.

We tested for heteroscedasticity using a Breusch-Pagan test (*bptest,* R package *lmtest)* and misspecification of the variance function by fitting a model that allows the precision (phi) to depend on the covariate (e.g., canopyArea~max_mean_temp | max_mean_temp) and comparing this to the simpler model using *lrtest* from *lmtest*. To test for spatial autocorrelation, we averaged scaled canopy area across years by zone and calculated Moran’s I for each sub-region (R package *ape*; [39]). We found no evidence of spatial autocorrelation among zones within each region (all p > 0.15). Finally, we compared AIC values among models to determine which temperature predictor produced the best-fit model.

## Results

### Floating canopy-forming kelp distribution and temporal variability

Floating kelp in Washington State exhibited substantial interannual variability (Tables 1; S1 and S2), with up to 13-fold range in canopy area at Cherry Point (Table 1). The region with the most canopy area was the Western Strait of Juan de Fuca, followed by the open coast and the Eastern Strait (Table 1). Smaller kelp bed areas in the three DNR Aquatic Reserves coincide with smaller surveyed area.

Canopy areas of *Nereocystis* and *Macrocystis* (2011–2018) were well correlated (r = 0.79, p < 0.001). However, the mean percentage of maximum canopy area observed (2011–2018) was lower (Wilcoxon rank sum test; W = 29036, p < 0.001) with more variability (Levene F = 53, p < 0.001) for *Nereocystis* (54 ± 29%; mean ± s.d.) than *Macrocystis* (77 ± 16%) (S2b Fig).

### Floating canopy-forming kelp loss and recovery

Region-wide decreases in kelp canopies coincided with the 2014–2016 MHW [10,11]. Sub-regions in this study experienced a maximum 36–85% loss compared to the preceding 3 years (Fig 1d; Table 1). However, recovery time varied, with sub-regions at or near the open ocean recovering more quickly than sub-regions further into the inland Salish Sea (Fig 1e, f). Specifically, the open coast and Strait of Juan de Fuca sub-regions recovered to the pre-warming mean by 2015 (Fig 1e), Smith and Minor Islands recovered by 2016, and Cypress Island and Cherry Point recovered by 2017 (Fig 1f).

### Variable temperature trajectories

**Fig 2:**
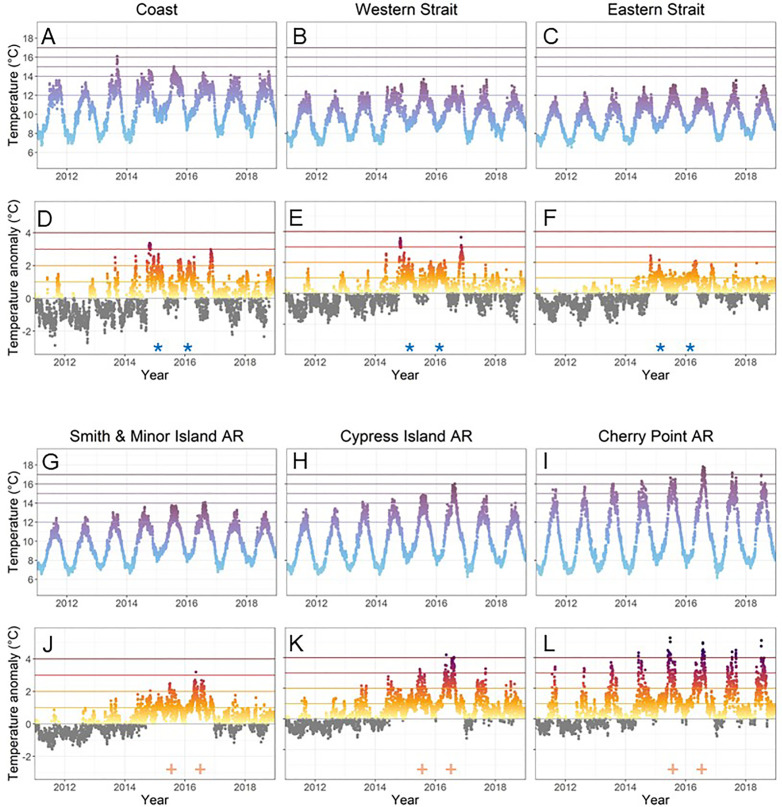
Daily mean sea surface temperature (SST, panels A-C and G-I) and daily mean SST anomaly (SSTA, panels D-F and J-L), for a representative zone in each sub-basin (columns). In the SST panels, horizontal lines are marked at 12°C, 14°C, 15°C, 16°C, and 17°C, and individual points are colored by SST. In the SSTA panels, horizontal lines corresponding to positive SSTA are placed at +1°C (yellow), + 2°C (orange), + 3°C (red), and +4°C (maroon), and individual points are colored gray when SSTA<0, and a gradient of yellow to maroon corresponding to SSTA values > 0. Blue asterisks (*) indicate periods of positive SSTA that occurred primarily during winter, and brown plus signs (+) indicate periods of positive SSTA that occurred primarily during summer. Daily mean sea surface temperatures of representative zones (panels A-C and G-I) are compared to all other zones in S3 Fig.

Sea surface temperature (SST) showed strong seasonal patterns that diverged across sub-regions (Fig 2; S3 and S4 Table). Minimum winter temperatures were notably higher across regions in 2015 and 2016 (Fig 2). Elevated temperatures occurred in summer and fall. The Strait sub-regions never exceeded 14°C daily mean SST during the study period, and Smith and Minor only briefly exceeded 14°C in 2016. In contrast, the open coast and Cypress Island exceeded 14°C yearly from 2013–2018, both also exceeded 16°C briefly (open coast in 2013, Cypress in 2016). Cherry Point was the warmest sub-region, exceeding 15°C in all years and exceeding 17°C in 2016 and 2017 (Fig 2).

SST anomalies diverged among sub-regions (Fig 2). Both the magnitude and duration of positive SSTA extremes were greatest at the inland sites, where there were only a handful of cooler days between 2015 and 2017 (Fig 2; S5 Table). The largest SSTAs on the open coast and Strait of Juan de Fuca occurred during winter (Fig 2b“*”), while the largest SSTAs for the inner sub-regions occurred during summer (Fig 2b“+”). Overall, maximum SSTAs were highest at Cypress Island and Cherry Point, and lowest in the Eastern Strait and Smith and Minor AR (Fig 2).

The number of days with SSTA>0°C varied among years and sub-regions (S6 Table). Before the MHW (mean 2011–2013), the fewest days with SSTA>0°C occurred in the Eastern Strait (64), Western Strait (75), and Smith and Minor (79), followed by the open coast and Cypress Island (94; 126), and the most at Cherry Point (170). During the MHW (mean 2015–2016), the number of days with SSTA>0°C was more similar among sub-regions, with slightly fewer days in the Western Strait (296), open coast (299), and Eastern Strait (320) compared to Cherry Point (358), Cypress Island (363), and Smith and Minor (364).

### Temperature metrics and canopy persistence

**Fig 3:**
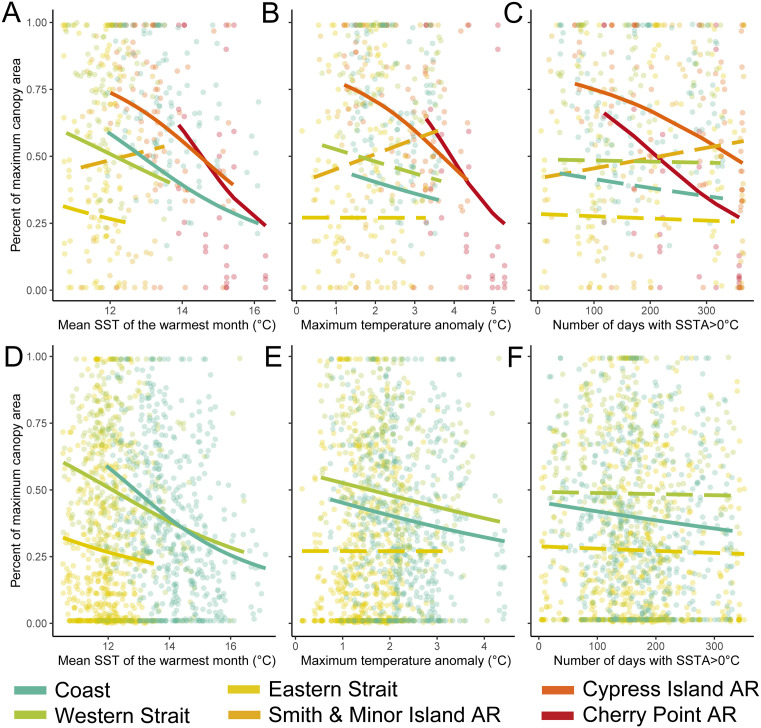
Relationships between temperature metrics and floating kelp area (defined as percent of maximum canopy area) from 2011-2018 (panels a-c) and from 1989-2021 (panels d-f). All panels represent total floating kelp canopy area, including both *Nereocystis luetkeana* and *Macrocystis pyrifera*. Fitted lines are from beta regressions by sub-region. Dashed lines indicate non-significant effects and solid lines indicate significant effects. Points show individual data points, with one data point representing one zone within a sub-region.

Four of six sub-regions exhibited a negative relationship between kelp canopy area and mean SST of the warmest month (2011−2018; Fig 3a; Table 2); this relationship explained 5−13% of variability in canopy area. In all statistically significant models, temperature metrics were negatively related to kelp canopy area. For the open coast, mean SST of the warmest month was the best-fit model (pseudo-R^2^ = 0.08), although the maximum temperature anomaly model was also significant (Fig 3a–c). In the Western Strait, only mean SST of the warmest month was significantly related to canopy area (pseudo-R^2^ = 0.05; Fig 3a–c). In the Eastern Strait and Smith and Minor Islands AR, none of the tested metrics were significantly related to canopy area (Fig 3a–c). At Cypress Island and Cherry Point, all three metrics were significantly related to canopy area (Fig 3a–c), with maximum temperature anomaly and number of days with SSTA>0°C the best-fit models (<1 ΔAIC; Table 2; Cypress Island pseudo-R^2^ = 0.11, Cherry Point pseudo-R^2^ = 0.18). A separate analysis of 1989−2021 for the coast and Strait sub-regions showed similar, but weaker, relationships (pseudo-R^2^ = 0.001–0.03; Fig 3d–f, Table 2).

**Table 2:** Temperature predictors with associated statistical results of beta regression models by sub-region and temperature metric for both species combined (corresponding to Fig 3a–c and 3d–f). For the Eastern Strait and Smith and Minor AR, none of the models were significant. Asterisks (*) represent cases where the Breusch-Pagan test identified residual heteroscedasticity, and φ represents a case where the misspecification of variance test was significant. All other models passed both of these tests for model fit.

Region	Predictor	2011-2018			1989-2021		
		p-value	pseudo-R^2^	AIC	p-value	pseudo-R^2^	AIC
Open Coast	Max. monthly mean temperature Max. mon. temperature anomaly Number of days with SSTA>0°C	<0.001 <0.001 0.06	0.08 0.05 0.02	−53.4 −50.4 −40.3	<0.001 0.007 0.044	0.03 0.003 0.003	−128.9 −89.2 −85.9
Western Strait	Max. monthly mean temperature Max. mon. temperature anomaly Number of days with SSTA>0°C	<0.001* >0.1 >0.1	0.05 – –	−52.2 – –	<0.001*φ 0.038 >0.1	0.01 0.001 –	−13.7 −1.0 –
Eastern Strait	Max. monthly mean temperature Max. mon. temperature anomaly Number of days with SSTA>0°C	>0.1 >0.1 >0.1	– – –	– – –	0.036 >0.1 >0.1	0.03 – –	−732.2 – –
Smith & Minor Island AR	Max. monthly mean temperature Max. mon. temperature anomaly Number of days with SSTA>0°C	>0.1 >0.1 >0.1	– – –	– – –	**–** **–** **–**	– – –	– – –
Cypress Island AR	Max. monthly mean temperature Max. mon. temperature anomaly Number of days with SSTA>0°C	0.009 0.002 0.002	0.05 0.11 0.11	−23.1 −27.7 −27.8	**–** **–** **–**	– – –	– – –
Cherry Point AR	Max. monthly mean temperature Max. mon. temperature anomaly Number of days with SSTA>0°C	0.04 0.01 0.02	0.13 0.18 0.18	−16.4 −18.8 −18.3	**–** **–** **–**	– – –	– – –

When divided by species in sub-regions where both species were commonly present (i.e., open coast and Western Strait; S7 Table) during 2011–2018, *Nereocystis* followed a similar pattern to overall kelp canopy, with the exception that mean SST of the warmest month was not significantly related to canopy area for the Western Strait (S4a–c Fig). *Macrocystis* followed the same pattern as overall kelp canopy (S4d–f Fig). Across the entire dataset (1989–2021), species patterns were similar to 2011–2018, except that *Nereocystis* on the Coast was not significantly related to maximum SSTA and that *Macrocystis* was significantly negatively related to maximum SSTA for the Western Strait (Fig S5; S8 Table).

## Discussion

The 2014–2016 MHW coincided with a rapid decline in kelp canopy abundance throughout the study region (−36–85%). However, trajectories varied dramatically among sub-regions. The open coast and Strait of Juan de Fuca returned to baseline abundance in one year (Fig 1d), showing a rapid recovery similar to Oregon [15]. In contrast, inland sub-regions experienced subsequent declines and delayed recovery; Smith and Minor Islands recovered after two years (Fig 1d), while the two furthest inland sub-regions (Cypress Island, Cherry Point) recovered after three years (Fig 1d).

While the temperature metrics provide insight into the relationship between temperature and kelp canopy area in Washington, all showed low explanatory power. For the three sub-basins where we have >30 years of data, all three temperature metrics were more strongly related to kelp canopy during 2011–2018 than during the full dataset (1989–2021; Fig 3, Table 2). This suggests that multiple environmental and biological drivers may mask temperature signals over the past three decades, but that temperature signals strengthened during the 2014–2016 MHW. During 2011–2018, mean SST of warmest month predicted floating canopy area over the most sub-regions (4 of 6), but only explained ≤13% of variability. At Cypress Island and Cherry Point, the warmest inner sub-regions in this study, magnitude (SSTA) and duration (days with SSTA>0°C) were the best-fit models, but they only explained ≤18% of variability. This points to a fundamental question: why did some locations (e.g., open coast and Strait) experience declines when exposed to only marginally warmer temperatures, while others (e.g., Cherry Point) were able to persist and recover despite longer duration and higher intensity warming?

One explanation could be that kelp dynamics were driven by nuances related to the timing of elevated SST. While the MHW lasted for two years, the timing of temperature anomalies varied among sub-regions (e.g., the largest anomalies on the coast and Strait occurred mostly in winter, and for the Aquatic Reserves they occurred primarily in summer; Fig 2). For this reason, we suggest that additional research is needed to assess timing of MHW in relation to potential ‘pinch points’ in the kelp lifecycle. For example, sori production of *N. luetkeana* peaks in mid-summer through Fall, although it may occur year round [40], while *Macrocystis* sori production is variable over time [41,42]. Additionally, variability might be due to species-specific responses; while temperature responses were similar between species (S4 and S5 Fig), we found more interannual variability in *Nereocystis* than *Macrocystis* (S2 Fig). Local adaptation may also play a role in kelp response. There are two main groups of *Nereocystis luetkeana* genetic co-ancestry in Washington, one along the open coast and Strait of Juan de Fuca and one within the inner Salish Sea [43]. It is possible that population structure could explain some differences between Cherry Point *Nereocystis* and *Nereocystis* from other sites, since it is geographically located between the two groups of genetic co-ancestry [43]. However, Cherry Point *Nereocystis* were not sampled in Gierke et al. 2023 [43], so additional research is needed to address this hypothesis. Although local adaptation could play a role in geographic variability, populations exposed to warmer conditions may not be more resilient to elevated temperatures [20,23].

At first glance, it appears that elevated temperatures could have driven declines on the open coast; the mean daily SST of 16.1°C in Fall 2013 (Fig 2) exceeded a proposed thermal threshold for *Nereocystis* [15]. The temperature spike occurred in Fall, which could have impacted multiple life stages, including the perennial giant kelp sporophyte and the sori or microscopic stages of both bull kelp and giant kelp. However, temperature alone was not likely to be the cause for two reasons: 1) the period of elevated temperatures was relatively short (~5 days; Fig 2); and 2) the Eastern and Western Strait sub-regions experienced similar losses, yet temperatures remained low (13.7°C; 11.8°C). Canopy kelp abundance in these areas is correlated with oceanic indices [28], suggesting potential interacting influences of large-scale variability in temperature, salinity, or nitrate concentrations on canopy persistence.

The most extreme temperatures and prolonged kelp declines were observed at Cherry Point. There, the persistence of canopy kelp contradicts many expectations. Kelp canopies at Cherry Point decreased significantly when the maximum monthly temperature was 14.6°C and recovered while the maximum monthly temperature was more extreme (15.4°C). The annual temperature record (Fig 2) showed extreme and persistent elevated temperatures (>16°C), >+4°C anomalies annually from 2014–2018. In addition to temperature stress, extremely low nutrient concentrations likely occurred; summer total N concentrations < 1uM have been observed in other years [18]. As in other regions, elevated temperatures and low nutrients are empirically correlated in Washington, and seasonal nutrient drawdowns accompany elevated temperatures [31]. However, it is difficult to disentangle the effects of elevated temperatures and nutrient concentrations on kelp canopies. More research is needed to understand why Cherry Point kelp canopies persist; this represents a regional question with broad-scale implications, since understanding persistence at marginal locations can directly inform decisions regarding habitat protection and restoration at larger scales.

Encouragingly, we found broad recovery of floating kelp canopies after the 2014–2016 MHW at all sub-regions in this study. However, these recoveries should be taken in the context of losses without recovery at other locations in Washington [31] and regionally (e.g., [8,44]). It is likely that that ongoing warming poses an immediate risk to kelp populations within the Salish Sea [31]. However, pressing questions remain regarding mechanisms of resistance and recovery. Continued work is needed to evaluate the spatial and temporal variability of biological responses to extreme climate events, like MHWs, to elucidate the potential vulnerabilities and resilience of our critical ecosystems to climate change.

## Supporting information

S1 Table Floating kelp bed area in six sub-regions in Washington State, USA. Bed area includes the area of floating kelp individuals at the surface of the water as well as the spaces between adjacent individuals. Surveys were conducted from 1989–2021 (excluding 1993) for the open coast and Strait of Juan de Fuca, and from 2011–2021 for the DNR Aquatic Reserves.
(DOCX)

S2 Table Floating kelp canopy area in six sub-regions in Washington State, USA. Canopy area includes the area of floating kelp individuals at the surface of the water only. Surveys were conducted from 1989–2021 (excluding 1993) for the open coast and Strait of Juan de Fuca, and from 2011–2021 for the DNR Aquatic Reserves.
(DOCX)

S3 Table Summary of temperature metrics for the representative zone in each sub-region (shown in Fig 2): maximum monthly mean sea surface temperature (SST) and maximum monthly mean SST anomaly (SSTA). Values include temperature metrics from September in the first year to August in the second year (e.g., September 2010 to August 2011).
(DOCX)

S4 Table Monthly maximum SST by sub-region (mean (minimum-maximum)). Values include temperature metrics from September in the first year to August in the second year (e.g., September 2010 to August 2011). Mean includes all zones within each sub-region. All zones of Cherry Point AR are included within one SST pixel.
(DOCX)

S5 Table Maximum monthly SST anomaly by sub-region (mean (minimum-maximum)). Values include temperature metrics from September in the first year to August in the second year (e.g., September 2010 to August 2011). Mean includes all zones within each sub-region. All zones of Cherry Point AR are included within one SST pixel.
(DOCX)

S6 Table Number of days with SSTA>0°C by sub-region (mean (minimum-maximum)). Values include temperature metrics from September in the first year to August in the second year (e.g., September 2010 to August 2011). Mean includes all zones within each sub-region. All zones of Cherry Point AR are included within one SST pixel.
(DOCX)

S7 Table Relationship between temperature metrics and floating kelp canopy area (defined as percent of maximum canopy area observed) for *Macrocystis* and *Nereocystis* in sub-regions where they commonly co-occur. Results of beta regression models by sub-region and temperature metric for years 2011–2018.
(DOCX)

S8 Table Relationship between temperature metrics and floating kelp canopy area (defined as percent of maximum canopy area observed) for *Macrocystis* and *Nereocystis* in sub-regions where they commonly co-occur. Results of beta regression models by sub-region and temperature metric for all years (1989–2021).
(DOCX)

S1 Fig Floating kelp canopy area, from low-tide aerial imagery collected between 1989 and 2021 (excluding 1993). Panel a shows total floating kelp area within each region for each year, and the gray line indicates the onset of the marine heatwave. Sub-panels in Panel b show details of floating kelp area for the three DNR Aquatic Reserves: Smith and Minor Island AR, Cypress Island AR, and Cherry Point AR.
(TIF)

S2 Fig A) Distribution of canopy-forming kelp species in the study area. Sub-basins are labeled, and short, solid black lines delineate the boundary between the two Strait sub-regions and the Coast sub-region. Only one zone contains exclusively *M. pyrifera* (inner Neah Bay, just East of the boundary between the Coast and Western Strait). B) Density plot showing the distribution of percent of maximum canopy area during each year and map index. *Nereocystis* has a wider distribution with a peak just below 50%, indicating that it fluctuates considerably between years. Conversely, *Macrocystis* has a narrower distribution with a peak around 75%, indicating that *Macrocystis* canopy area is more consistent over time. Map credits: Commission for Environmental Cooperation, Statistics Canada 2022.
(TIF)

S3 Fig Daily mean sea surface temperature (SST) from 2011–2018, comparing representative zones (depicted in Figure 2) in black, and all other zones within each sub-region in gray. Horizontal lines represent potential thresholds: 14°C (gray), 15°C (yellow), 16°C (orange), 17°C (red), 18°C (maroon).
(TIF)

S4 Fig Relationships between temperature metrics and floating kelp area (defined as percent of maximum canopy area) from 2011–2018 divided by canopy-forming species. Panels a-c and d-f represent canopy cover of *N. luetkeana* only and *M. pyrifera* only, respectively, in the two sub-regions where they commonly co-occur (Coast and Western Strait). Fitted lines are from beta regressions by sub-region. Dashed lines indicate non-significant effects and solid lines indicate significant effects. Points show individual data points, with one data point representing one zone within a sub-region.
(TIF)

S5 Fig Relationships between temperature metrics and floating kelp area (defined as percent of maximum canopy area) from 1989–2021 divided by canopy-forming species. Panels a-c and d-f represent canopy cover of *N. luetkeana* only and *M. pyrifera* only, respectively, in the two sub-regions where they commonly co-occur (Coast and Western Strait). Fitted lines are from beta regressions by sub-region. Dashed lines indicate non-significant effects and solid lines indicate significant effects. Points show individual data points, with one data point representing one zone within a sub-region.
(TIF)
