# Supplementary material for: Geographic variability of floating kelp recovery after a marine heatwave event in the Salish Sea and adjacent open coast
Source: PLoS One. 2025 Dec 2;20(12):e0336574. doi: 10.1371/journal.pone.0336574 (PMC12671756; doi:10.1371/journal.pone.0336574)
Supplement: S1 Table — Bed area includes the area of floating kelp individuals at the surface of the water as well as the spaces between adjacent individuals. Surveys were conducted from 1989–2021 (excluding 1993) for the open coast and Strait of Juan de Fuca, and from 2011–2021 for the DNR Aquatic Reserves. (DOCX) [file pone.0336574.s001.docx]

Table S1. Floating kelp bed area in six sub-regions in Washington State, USA. Bed area includes the area of floating kelp individuals at the surface of the water as well as the spaces between adjacent individuals. Surveys were conducted from 1989-2021 (excluding 1993) for the open coast and Strait of Juan de Fuca, and from 2011-2021 for the DNR Aquatic Reserves.

| Sub-region | Years included | Min. bed area (ha) | Mean bed area (ha) | Max. bed area (ha) | Fold difference between min. and max. bed area |
| --- | --- | --- | --- | --- | --- |
| Open Coast | 1989-2021 | 475 | 983 ± 321 | 1725 | 3.6 |
| Western Strait | 1989-2021 | 1561 | 2433 ± 458 | 3625 | 2.3 |
| Eastern Strait | 1989-2021 | 349 | 824 ± 350 | 1833 | 5.3 |
| Smith & Minor Islands AR | 2011-2021 | 174 | 366 ± 199 | 856 | 4.9 |
| Cypress Island AR | 2011-2021 | 55 | 67 ± 8 | 79 | 1.5 |
| Cherry Point AR | 2011-2021 | 33 | 85 ±29 | 118 | 3.8 |
