# Supplementary material for: Geographic variability of floating kelp recovery after a marine heatwave event in the Salish Sea and adjacent open coast
Source: PLoS One. 2025 Dec 2;20(12):e0336574. doi: 10.1371/journal.pone.0336574 (PMC12671756; doi:10.1371/journal.pone.0336574)
Supplement: S2 Table — Canopy area includes the area of floating kelp individuals at the surface of the water only. Surveys were conducted from 1989–2021 (excluding 1993) for the open coast and Strait of Juan de Fuca, and from 2011–2021 for the DNR Aquatic Reserves. (DOCX) [file pone.0336574.s002.docx]

Table S2. Floating kelp canopy area in six sub-regions in Washington State, USA. Canopy area includes the area of floating kelp individuals at the surface of the water only. Surveys were conducted from 1989-2021 (excluding 1993) for the open coast and Strait of Juan de Fuca, and from 2011-2021 for the DNR Aquatic Reserves.

| Sub-region | Years included | Min. canopy area (ha) | Mean canopy area (ha) | Max. canopy area (ha) | Fold difference between min. and max. canopy area |
| --- | --- | --- | --- | --- | --- |
| Open Coast | 1989-2021 | 142 | 329 ± 116 | 521 | 3.7 |
| Western Strait | 1989-2021 | 442 | 945 ± 291 | 1643 | 3.7 |
| Eastern Strait | 1989-2021 | 73 | 232 ± 120 | 518 | 7.1 |
| Smith & Minor Islands AR | 2011-2021 | 38 | 126 ± 100 | 382 | 10.2 |
| Cypress Island AR | 2011-2021 | 9.3 | 13 ± 2 | 15 | 1.6 |
| Cherry Point AR | 2011-2021 | 2.3 | 16.5 ± 10 | 30 | 13 |
