# Supplementary material for: Geographic variability of floating kelp recovery after a marine heatwave event in the Salish Sea and adjacent open coast
Source: PLoS One. 2025 Dec 2;20(12):e0336574. doi: 10.1371/journal.pone.0336574 (PMC12671756; doi:10.1371/journal.pone.0336574)
Supplement: S3 Table — Values include temperature metrics from September in the first year to August in the second year (e.g., September 2010 to August 2011). (DOCX) [file pone.0336574.s003.docx]

Table S3. Summary of temperature metrics for the representative zone in each sub-region (shown in Figure 2): maximum monthly mean sea surface temperature (SST) and maximum monthly mean SST anomaly (SSTA). Values include temperature metrics from September in the first year to August in the second year (e.g., September 2010 to August 2011).

| Sub-region | Temperature metric | 2010-2011 | 2011-2012 | 2012-2013 | 2013-2014 | 2014-2015 | 2015-2016 | 2016-2017 | 2017-2018 |
| --- | --- | --- | --- | --- | --- | --- | --- | --- | --- |
| Open Coast | Max. mon. SST (°C) | 12.2 | 12.5 | 12.7 | 14.2 | 13.9 | 13.3 | 12.7 | 12.7 |
|  | Max. mon. SSTA (°C) | 1.99 | 1.47 | 1.49 | 2.57 | 3.36 | 2.52 | 2.98 | 1.64 |
|  | Days with SSTA>0°C | 71 | 39 | 89 | 118 | 273 | 279 | 178 | 163 |
| Western Strait | Max. mon. SST (°C) | 11.1 | 11.3 | 11.6 | 11.9 | 12.5 | 12.1 | 11.8 | 11.6 |
|  | Max. mon. SSTA (°C) | 2.02 | 1.58 | 1.35 | 2.55 | 3.55 | 2.12 | 3.62 | 1.84 |
|  | Days with SSTA>0°C | 86 | 43 | 112 | 122 | 302 | 303 | 174 | 173 |
| Eastern Strait | Max. mon. SST (°C) | 10.7 | 10.9 | 11.4 | 11.7 | 12.2 | 11.9 | 11.9 | 12.0 |
|  | Max. mon. SSTA (°C) | 0.82 | 0.44 | 0.86 | 1.07 | 2.43 | 2.08 | 1.86 | 1.96 |
|  | Days with SSTA>0°C | 73 | 11 | 97 | 90 | 323 | 310 | 208 | 212 |
| Smith & Minor AR | Max. mon. SST (°C) | 11.5 | 12.0 | 11.4 | 12.4 | 13.0 | 13.1 | 12.0 | 12.1 |
|  | Max. mon. SSTA (°C) | 0.79 | 1.42 | 1.06 | 1.82 | 2.49 | 3.19 | 1.68 | 1.76 |
|  | Days with SSTA>0°C | 25 | 118 | 106 | 171 | 362 | 366 | 239 | 226 |
| Cypress Island AR | Max. mon. SST (°C) | 12.5 | 13.0 | 12.1 | 13.1 | 13.9 | 14.9 | 12.9 | 12.8 |
|  | Max. mon. SSTA (°C) | 1.31 | 2.16 | 1.36 | 2.76 | 3.24 | 4.17 | 2.52 | 3.27 |
|  | Days with SSTA>0°C | 70 | 170 | 129 | 199 | 364 | 362 | 291 | 316 |
| Cherry Point AR | Max. mon. SST (°C) | 14.1 | 15.0 | 13.9 | 14.6 | 15.2 | 16.3 | 15.4 | 15.2 |
|  | Max. mon. SSTA (°C) | 3.29 | 3.68 | 3.4 | 4.31 | 5.28 | 4.97 | 4.37 | 5.11 |
|  | Days with SSTA>0°C | 118 | 222 | 171 | 217 | 358 | 358 | 287 | 317 |
