# Supplementary material for: Geographic variability of floating kelp recovery after a marine heatwave event in the Salish Sea and adjacent open coast
Source: PLoS One. 2025 Dec 2;20(12):e0336574. doi: 10.1371/journal.pone.0336574 (PMC12671756; doi:10.1371/journal.pone.0336574)
Supplement: S4 Table — Values include temperature metrics from September in the first year to August in the second year (e.g., September 2010 to August 2011). Mean includes all zones within each sub-region. All zones of Cherry Point AR are included within one SST pixel. (DOCX) [file pone.0336574.s004.docx]

Table S4. Monthly maximum SST by sub-region (mean (minimum-maximum)). Values include temperature metrics from September in the first year to August in the second year (e.g., September 2010 to August 2011). Mean includes all zones within each sub-region. All zones of Cherry Point AR are included within one SST pixel.

|  | 2010-2011 | 2011-2012 | 2012-2013 | 2013-2014 | 2014-2015 | 2015-2016 | 2016-2017 | 2017-2018 |
| --- | --- | --- | --- | --- | --- | --- | --- | --- |
| Open Coast | 12.9  (12.2-13.3) | 13.1  (12.1-13.7) | 15.2  (13.7-16.1) | 14.4  (13.4-14.9) | 14.5  (13.6-15.0) | 13.8  (12.8-14.5) | 13.1  (12.4-13.4) | 13.2 (12.6-13.7) |
| Western Strait | 11.3  (10.8-12.1) | 11.5  (11.0-12.1) | 12.2  (11.4-13.7) | 12.3  (11.7-13.3) | 12.7  (12.3-13.5) | 12.2  (11.9-12.7) | 11.9  (11.7-12.3) | 11.8  (11.4-12.5) |
| Eastern Strait | 10.7  (10.7-10.9) | 10.9 (10.9-11.1) | 11.4 (11.3-11.8) | 11.7  (11.6-12.1) | 12.3  (12.2-12.5) | 11.9  (11.9-12.2) | 11.9  (11.7-12.0) | 11.8  (11.4-12.0) |
| Smith & Minor AR | 11.1 (10.9-11.3) | 11.5 (11.2-11.6) | 12.0  (11.8-12.3) | 12.5  (12.2-12.7) | 13.0  (12.7-13.3) | 13.1  (12.6-13.5) | 12.1  (11.9-12.3) | 12.3  (12.1-12.5) |
| Cypress Island AR | 12.2  (12.0-12.4) | 12.7  (12.5-12.9) | 13.2  (13.0-13.5) | 13.2  (13.1-13.5) | 14.1  (13.9-14.3) | 15.2  (14.9-15.4) | 13.6  (12.9-13.9) | 13.1  (12.8-13.4) |
| Cherry Point AR | 13.9 | 14.1 | 15.0 | 14.6 | 15.2 | 16.3 | 15.4 | 15.2 |
