# Supplementary material for: Geographic variability of floating kelp recovery after a marine heatwave event in the Salish Sea and adjacent open coast
Source: PLoS One. 2025 Dec 2;20(12):e0336574. doi: 10.1371/journal.pone.0336574 (PMC12671756; doi:10.1371/journal.pone.0336574)
Supplement: S5 Table — Values include temperature metrics from September in the first year to August in the second year (e.g., September 2010 to August 2011). Mean includes all zones within each sub-region. All zones of Cherry Point AR are included within one SST pixel. (DOCX) [file pone.0336574.s005.docx]

Table S5. Maximum monthly SST anomaly by sub-region (mean (minimum-maximum)). Values include temperature metrics from September in the first year to August in the second year (e.g., September 2010 to August 2011). Mean includes all zones within each sub-region. All zones of Cherry Point AR are included within one SST pixel.

|  | 2010-2011 | 2011-2012 | 2012-2013 | 2013-2014 | 2014-2015 | 2015-2016 | 2016-2017 | 2017-2018 |
| --- | --- | --- | --- | --- | --- | --- | --- | --- |
| Open Coast | 1.9 (1.65-2.1) | 1.63 (1.4-1.81) | 2.01 (1.44-2.63) | 3.09 (2.54-3.52) | 3.44 (3.3-3.61) | 2.67 (2.44-2.91) | 2.94 (2.82-3.01) | 2.02 (1.61-2.35) |
| Western Strait | 2.08 (1.9-2.22) | 1.38 (0.65-1.65) | 1.36 (1.09-1.54) | 2.48 (2.16-2.63) | 3.54 (3.34-3.67) | 2.26 (1.95-2.57) | 3.21 (2.97-3.62) | 1.84 (1.5-2.19) |
| Eastern Strait | 1.11 (0.81-1.85) | 0.457 (0.14-0.56) | 0.923 (0.75-1.03) | 1.35 (1.03-2.01) | 2.71 (2.24-3.28) | 2.09 (1.99-2.38) | 2.16 (1.84-3.03) | 2.04 (1.59-2.24) |
| Smith & Minor AR | 1.12 (1.02-1.24) | .0631 (0.44-0.82) | 1.32 (1.11-1.43) | 1.72 (1.51-1.82) | 2.41 (2.15-2.57) | 3.18 (2.71-3.59) | 1.71 (1.68-1.75) | 1.76 (1.6-2.03) |
| Cypress Island AR | 1.44 (1.28-1.69) | 1.40 (1.21-1.69) | 2.24 (2.15-2.42) | 2.93 (2.76-3.19) | 3.37 (3.24-3.6) | 4.23 (4.17-4.34) | 3.07 (2.52-3.46) | 3.56 (3.27-3.75) |
| Cherry Point AR | 3.4 | 3.29 | 3.68 | 4.31 | 5.28 | 4.97 | 4.37 | 5.11 |
