# Supplementary material for: Geographic variability of floating kelp recovery after a marine heatwave event in the Salish Sea and adjacent open coast
Source: PLoS One. 2025 Dec 2;20(12):e0336574. doi: 10.1371/journal.pone.0336574 (PMC12671756; doi:10.1371/journal.pone.0336574)
Supplement: S6 Table — Values include temperature metrics from September in the first year to August in the second year (e.g., September 2010 to August 2011). Mean includes all zones within each sub-region. All zones of Cherry Point AR are included within one SST pixel. (DOCX) [file pone.0336574.s006.docx]

Table S6. Number of days with SSTA>0°C by sub-region (mean (minimum-maximum)). Values include temperature metrics from September in the first year to August in the second year (e.g., September 2010 to August 2011). Mean includes all zones within each sub-region. All zones of Cherry Point AR are included within one SST pixel.

|  | Annual values | | | | | | | | Time-period averages | |
| --- | --- | --- | --- | --- | --- | --- | --- | --- | --- | --- |
|  | 2010-2011 | 2011-2012 | 2012-2013 | 2013-2014 | 2014-2015 | 2015-2016 | 2016-2017 | 2017-2018 | 2010-2013 | 2014-2016 |
| Open Coast | 107 (71-149) | 68 (39-108) | 108 (88-139) | 143 (109-166) | 299 (269-329) | 299 (275-320) | 213 (166-250) | 183 (161-200) | 94 | 299 |
| Western Strait | 78 (72-86) | 43 (36-49) | 104 (91-118) | 121 (102-137) | 296 (273-325) | 296 (276-314) | 180 (166-208) | 170 (155-188) | 75 | 296 |
| Eastern Strait | 76 (72-87) | 16 (6-34) | 100 (70-111) | 104 (89-135) | 325 (313-350) | 315 (305-342) | 215 (201-255) | 208 (199-221) | 64 | 320 |
| Smith & Minor AR | 102 (91-106) | 21 (13-25) | 114 (92-131) | 168 (140-186) | 362 (355-365) | 365 (361-366) | 247 (230-277) | 244 (210-289) | 79 | 364 |
| Cypress Island AR | 131 (129-132) | 71 (66-78) | 175 (170-182) | 201 (199-202) | 363 (363-364) | 362 (360-363) | 292 (288-298) | 327 (316-332) | 126 | 363 |
| Cherry Point AR | 171 | 118 | 222 | 217 | 358 | 358 | 287 | 317 | 170 | 358 |
