# Supplementary material for: Geographic variability of floating kelp recovery after a marine heatwave event in the Salish Sea and adjacent open coast
Source: PLoS One. 2025 Dec 2;20(12):e0336574. doi: 10.1371/journal.pone.0336574 (PMC12671756; doi:10.1371/journal.pone.0336574)
Supplement: S7 Table — Results of beta regression models by sub-region and temperature metric for years 2011–2018. (DOCX) [file pone.0336574.s007.docx]

Table S7. Relationship between temperature metrics and floating kelp canopy area (defined as percent of maximum canopy area observed) for *Macrocystis* and *Nereocystis* in sub-regions where they commonly co-occur. Results of beta regression models by sub-region and temperature metric for years 2011-2018.

| Sub-region | Predictor | p-value | pseudo-R^2^ | AIC | p-value | pseudo-R^2^ | AIC |
| --- | --- | --- | --- | --- | --- | --- | --- |
|  |  | *Nereocystis* | | | *Macrocystis* | | |
| Open Coast | Max. monthly mean temperature | <0.001 | 0.06 | -13.0 | 0.026 | 0.04 | -70.5 |
|  | Max. mon. temperature anomaly | 0.006 | 0.05 | -7.3 | 0.023 | 0.09 | -72.7 |
|  | Number of days with SSTA>0°C | >0.1 | - | - | >0.1 | - | - |
| Western Strait | Max. monthly mean temperature | 0.07 | 0.05 | -13.7 | 0.009 | <0.01 | -85.9 |
|  | Max. mon. temperature anomaly | >0.1 | - | - | >0.1 | - | - |
|  | Number of days with SSTA>0°C | >0.1 | - | - | >0.1 | - | - |
