# Supplementary material for: Geographic variability of floating kelp recovery after a marine heatwave event in the Salish Sea and adjacent open coast
Source: PLoS One. 2025 Dec 2;20(12):e0336574. doi: 10.1371/journal.pone.0336574 (PMC12671756; doi:10.1371/journal.pone.0336574)
Supplement: S8 Table — Results of beta regression models by sub-region and temperature metric for all years (1989–2021). (DOCX) [file pone.0336574.s008.docx]

Table S8. Relationship between temperature metrics and floating kelp canopy area (defined as percent of maximum canopy area observed) for *Macrocystis* and *Nereocystis* in sub-regions where they commonly co-occur. Results of beta regression models by sub-region and temperature metric for all years (1989-2021).

| Sub-region | Predictor | p-value | pseudo-R^2^ | AIC | p-value | pseudo-R^2^ | AIC |
| --- | --- | --- | --- | --- | --- | --- | --- |
|  |  | *Nereocystis* | | | *Macrocystis* | | |
| Open Coast | Max. monthly mean temperature | <0.001 | 0.05 | -368.6 | 0.01 | 0.005 | -257.6 |
|  | Max. mon. temperature anomaly | >0.1 | - | - | 0.006 | 0.010 | -258.4 |
|  | Number of days with SSTA>0°C | 0.078 | 0.003 | -319.2 | >0.1 | - | - |
| Western Strait | Max. monthly mean temperature | >0.1 | - | - | <0.001 | 0.06 | -46.1 |
|  | Max. mon. temperature anomaly | 0.085 | 0.001 | 0.3 | <0.001 | 0.02 | -9.6 |
|  | Number of days with SSTA>0°C | >0.1 | - | - | >0.1 | - | - |
