# Supplementary figures and images for: Geographic variability of floating kelp recovery after a marine heatwave event in the Salish Sea and adjacent open coast
Source: PLoS One. 2025 Dec 2;20(12):e0336574. doi: 10.1371/journal.pone.0336574 (PMC12671756; doi:10.1371/journal.pone.0336574)

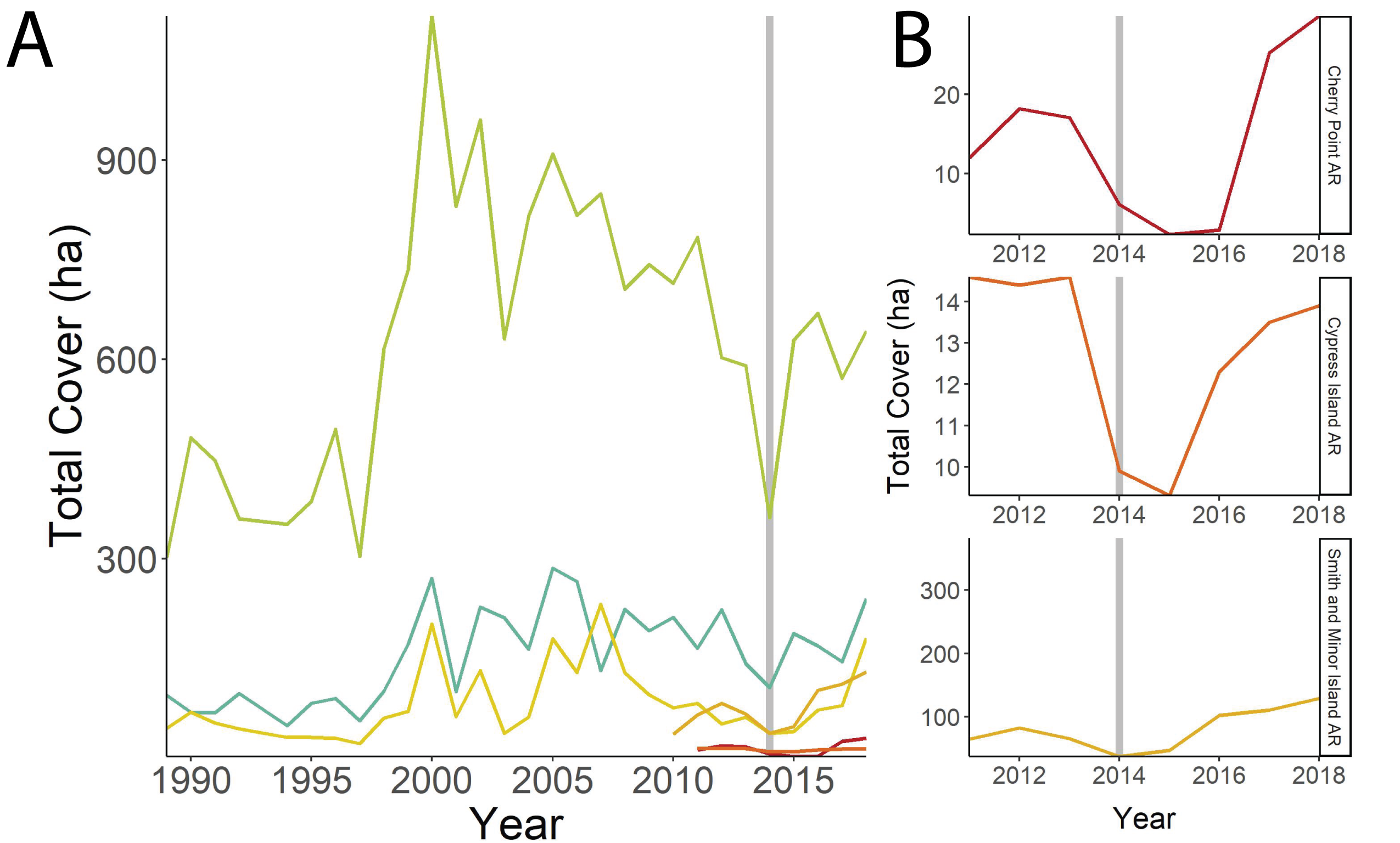

Supplement: S1 Fig — Panel a shows total floating kelp area within each region for each year, and the gray line indicates the onset of the marine heatwave. Sub-panels in Panel b show details of floating kelp area for the three DNR Aquatic Reserves: Smith and Minor Island AR, Cypress Island AR, and Cherry Point AR. (TIF) [file pone.0336574.s009.tif]

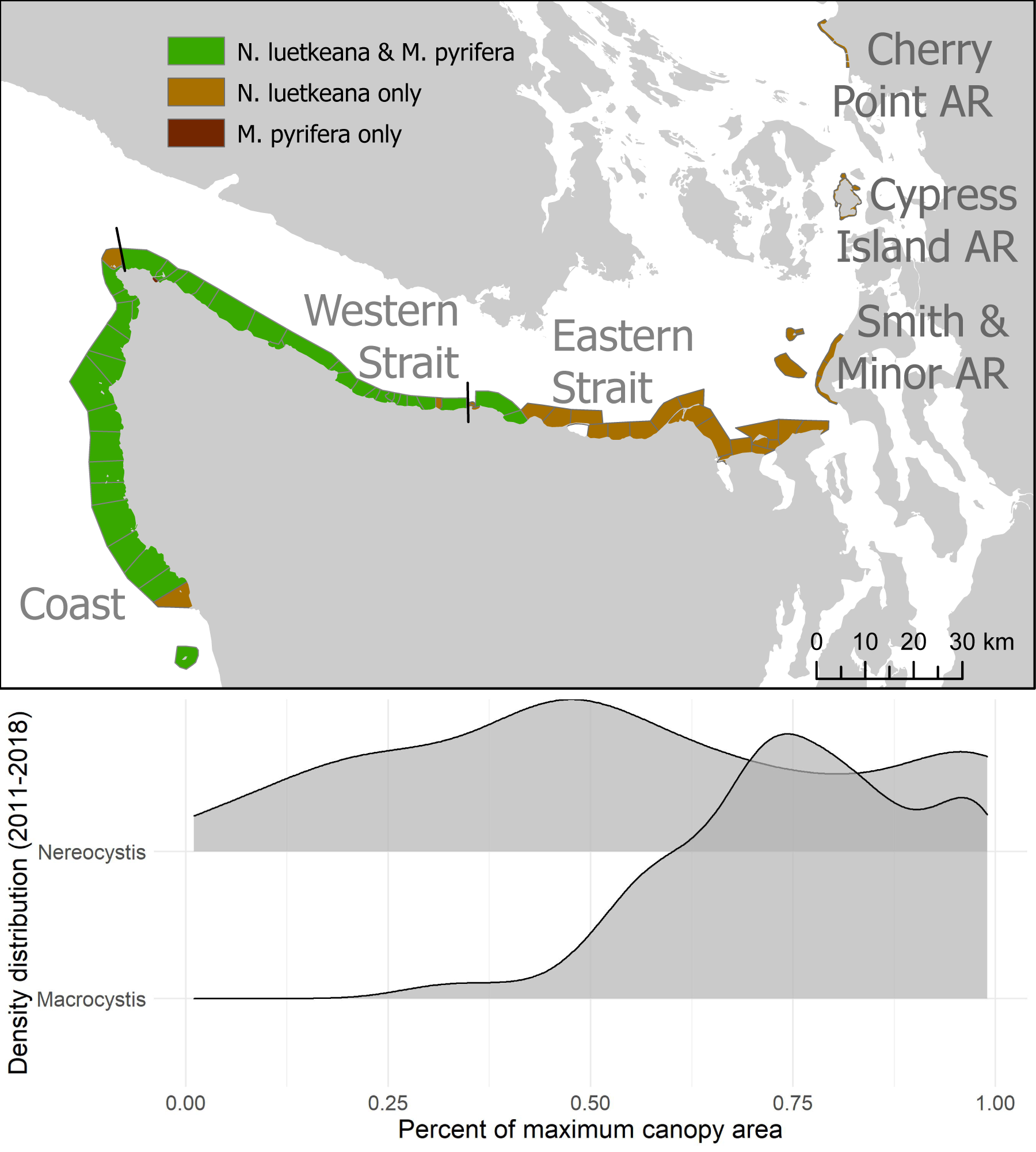

Supplement: S2 Fig — Sub-basins are labeled, and short, solid black lines delineate the boundary between the two Strait sub-regions and the Coast sub-region. Only one zone contains exclusively M. pyrifera (inner Neah Bay, just East of the boundary between the Coast and Western Strait). B) Density plot showing the distribution of percent of maximum canopy area during each year and map index. Nereocystis has a wider distribution with a peak just below 50%, indicating that it fluctuates considerably between years. Conversely, Macrocystis has a narrower distribution with a peak around 75%, indicating that Macrocystis canopy area is more consistent over time. Map credits: Commission for Environmental Cooperation, Statistics Canada 2022. (TIF) [file pone.0336574.s010.tif]

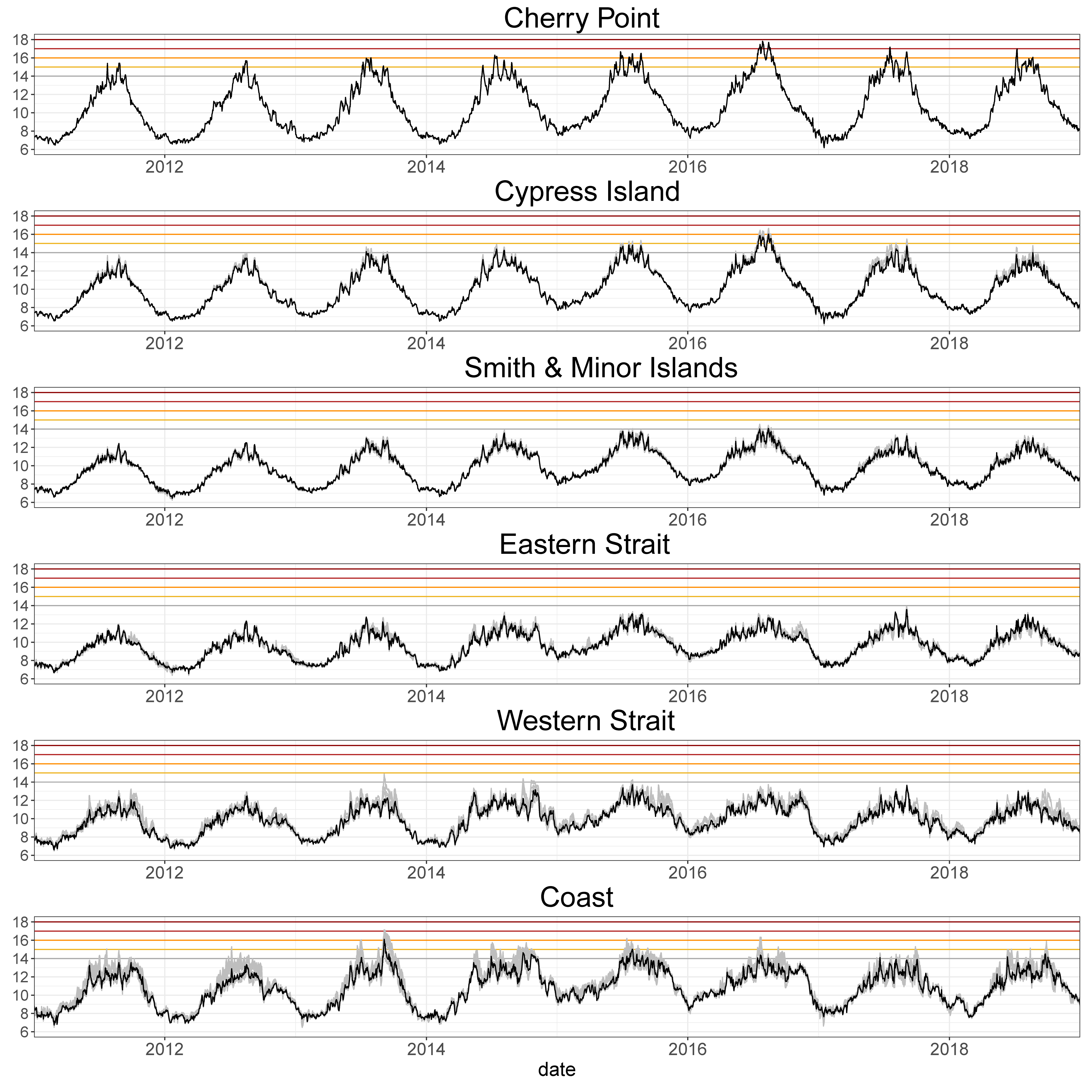

Supplement: S3 Fig — Horizontal lines represent potential thresholds: 14°C (gray), 15°C (yellow), 16°C (orange), 17°C (red), 18°C (maroon). (TIF) [file pone.0336574.s011.tif]

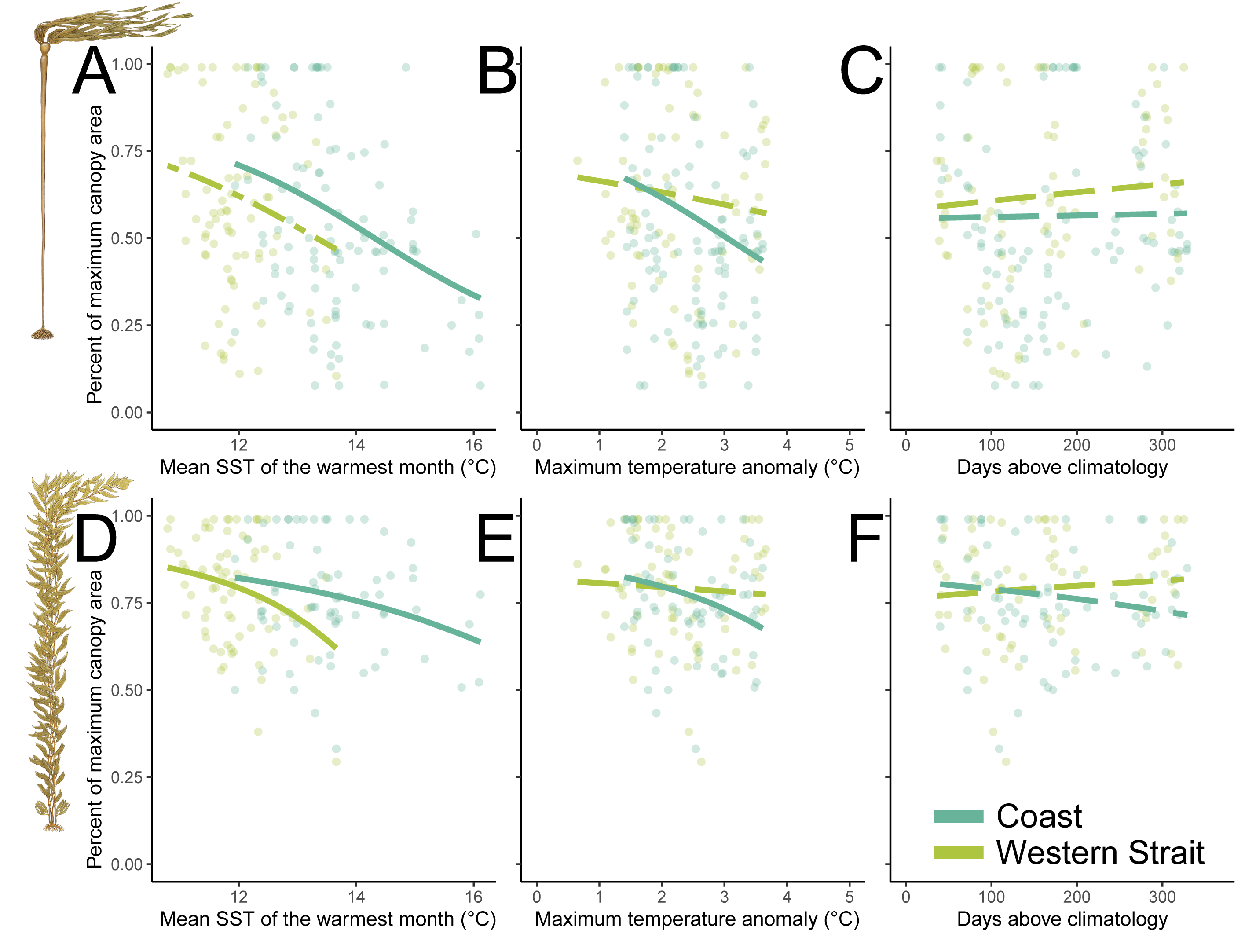

Supplement: S4 Fig — Panels a-c and d-f represent canopy cover of N. luetkeana only and M. pyrifera only, respectively, in the two sub-regions where they commonly co-occur (Coast and Western Strait). Fitted lines are from beta regressions by sub-region. Dashed lines indicate non-significant effects and solid lines indicate significant effects. Points show individual data points, with one data point representing one zone within a sub-region. (TIF) [file pone.0336574.s012.tif]

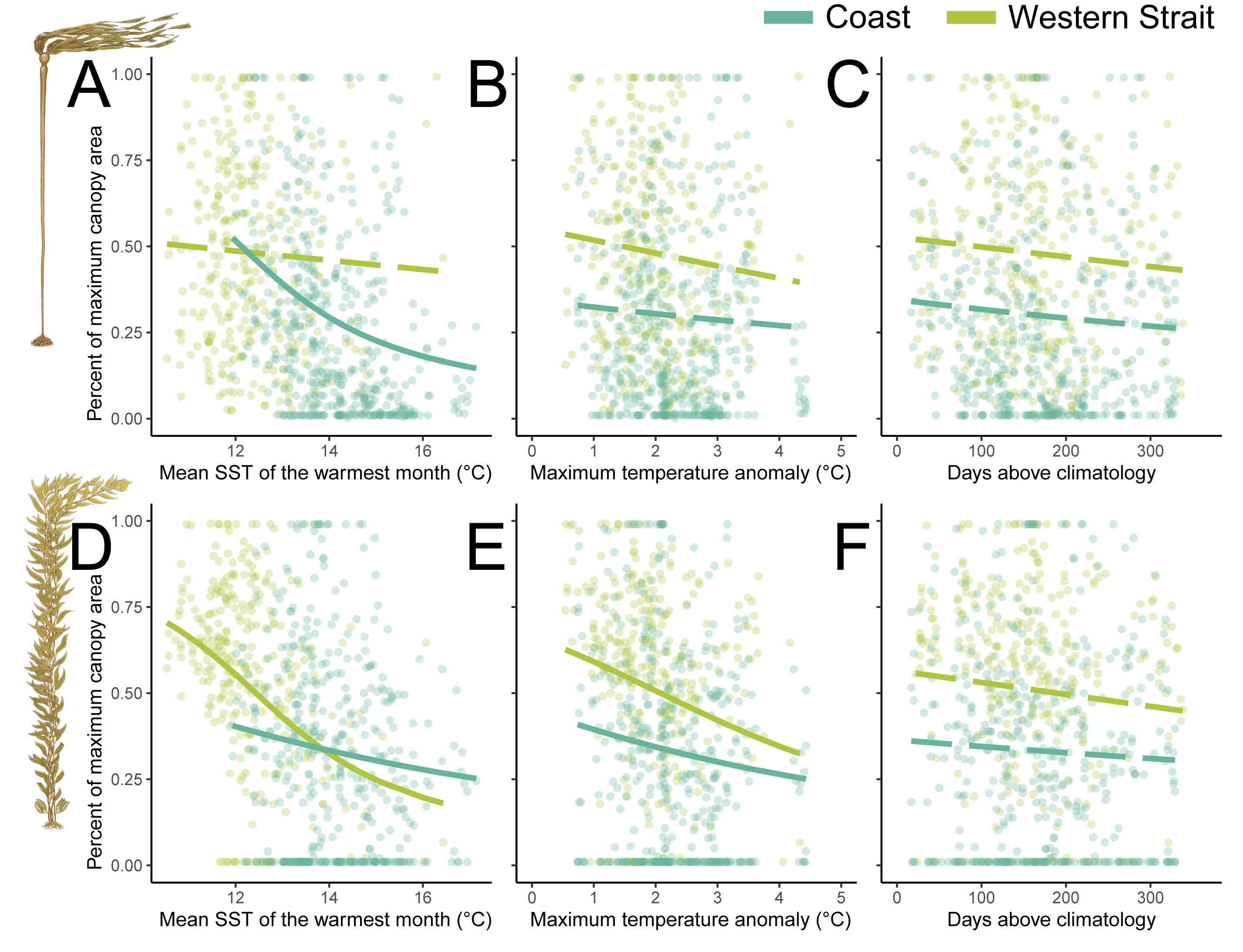

Supplement: S5 Fig — Panels a-c and d-f represent canopy cover of N. luetkeana only and M. pyrifera only, respectively, in the two sub-regions where they commonly co-occur (Coast and Western Strait). Fitted lines are from beta regressions by sub-region. Dashed lines indicate non-significant effects and solid lines indicate significant effects. Points show individual data points, with one data point representing one zone within a sub-region. (TIF) [file pone.0336574.s013.tif]
